# Mastery of type 2 diabetes prevention and treatment knowledge by general practitioners in Shanghai: a cross-sectional study

**DOI:** 10.1186/s12875-021-01538-1

**Published:** 2021-09-17

**Authors:** Yaling Li, Yunyun Yan, Huimin Dai, Yuan Cheng, Qian Huang, Jie Shao, Jianmin Zhou, Haitang Wang, Pingyang Liu, Ao Shen, Yikai Mi, Zhaohui Du

**Affiliations:** 1 Weifang Community Healthcare Center of Pudong New Area, Shanghai, China; 2 Zhongshan Hospital, Fudan University, Shanghai, China; 3 Shanggang Community Healthcare Center of Pudong New Area, Shanghai, China; 4 Health Dynamics Laboratory, School of Public Health, Shanghai Jiao Tong University, Shanghai, China

**Keywords:** General practitioners, Type 2 diabetes, Prevention and treatment knowledge, Survey

## Abstract

**Background:**

To study the competency of general practitioners (GPs) in Shanghai, China on prevention and management of type 2 diabetes, also understand factors that may prohibit it.

**Methods:**

A survey questionnaire with 25 questions was designed based on 2013 Chinese Type 2 Diabetes Prevention Guidelines and Chinese Type 2 Diabetes Prevention Guidelines (Grassroots Edition) and conducted among 789 GPs who work at 54 community healthcare centers (CHCs) within 16 districts at Shanghai, China. Excel 2016 and SPSS 24.0 were used for data analysis, and a difference of *P* < 0.05 was considered to be statistically significant.

**Results:**

The GPs did poorly on three aspect of diabetes prevention and treatment: (1) treatment goals in elderly patients, (2) screening methods for high-risk population, and (3) aspirin contraindications. The statistical analysis data showed that GPs who finished standardized training had correct answer on 13.58 ± 3.31 questions out of total 25, with mean accuracy rate of 54.32%. Except the questions for high-risk population screening method and the diagnostic criteria for type 2 diabetes, there was no difference in the accuracy of other questions between GPs with or without standardized training (*P* < 0.05). However, sex, educational level, and subspecialty experience are affective factors on their competency in type 2 diabetes prevention and treatment knowledge.

**Conclusion:**

The results indicated that communities should strengthen the training of GPs in diabetes management and bidirectional referral. Frequent continuing education and skills training should be provided among GPs at CHCs to ensure their competency of type 2 diabetes prevention and treatment knowledge after obtaining their GP license disregard of their standardized training. In addition, attention should be paid to GPs who had lower education background or non-clinical subspecialty experience to strengthen their clinical knowledge of type 2 diabetes.

## Background

Globally, diabetes places a significant burden both on the patients and society. In the United Kingdom, 7% of the population were living with diabetes in 2019 [1]. Meanwhile, in the United States, diabetes risk increases with time. The diabetes population would be doubled in 2050 than the population with diabetes in 2005 [2]. Similarly, in China, the prevalence of diabetes has increased rapidly. The epidemiological survey of 300,000 people in 14 provinces and cities in 1980 showed that the prevalence of diabetes was 0.67% [3]. While in 2017, the epidemiological survey of 31 provinces showed that the prevalence of diabetes among adults in China is 11.2% [4]. Obviously, diabetes is an important public health problem across the world, especially in China.

Traditionally, diabetes care was mainly promoted by endocrinologists at second-class or top first-class hospitals. In recent decades, diabetes care converted from high class hospitals to primary care internationally [5]. With the development of general practice in China, general practitioners (GPs) were involved in the management of chronic diseases including diabetes mellitus [6]. In 2015, the General Office of the State Council issued the guidelines for the construction of the graded diagnosis and treatment system, which emphasized the importance of chronic diseases managing by GPs at PHC facilities and bidirectional referral between primary care and high-class hospitals [7]. As the reform measures performed on the management of patients with chronic disease in the primary care gradually, contracted family doctor services were recommended in the PHC facilities nationally [8].

General medicine, or general practice, a clinical secondary discipline which has a shorter history of development for approximately 30 years in China [9]. The registered GPs in China were mainly from job-transfer training and “5 + 3” training. The job-transfer training referred to standardized training carried out for at least 12 months, on assistant medical practitioner of PHC facilities, certified doctor of primary or secondary care hospitals who were willing to be registered GPs [10]; the “5 + 3” training referred to 5 years of undergraduate study in medicine and 3 years of standardized training for clinical residents [9]. Shanghai as one of the representative pilot areas for general practice in China, its experience in developing general medicine was innovative, which would be popularized across the country [11]. However, although there were guidelines, homogeneous diabetes management has yet to be achieved at the PHC facilities [12].

As the GPs in China were not trained in the same way [9, 10], and the quality monitoring indicators for diabetes management were different among the PHC facilities [12], this research aimed to investigate the proficiency of type 2 diabetes prevention and treatment knowledge among GPs from PHC facilities in Shanghai and analyze the factors that affected this proficiency. Based on the results, targeted construction of quality monitoring indicators and relative training on the knowledge for GPs in the management for diabetes mellitus could be performed and further be used by the GPs at PHC facilities all over the country.

## Materials and methods

In this study, the General Practitioners’ Mastery of Chinese Type 2 Diabetes Prevention Guidelines questionnaire was used to investigate the proficiency of type 2 diabetes prevention and treatment knowledge in GPs in Shanghai and to examine relevant factors that affect the proficiency of type 2 diabetes prevention and treatment knowledge in GPs.

### Subjects

Simple randomization sampling was employed in this study to select 789 GPs from CHCs in Shanghai that conduct standardized general practitioner training (accredited by the Shanghai Municipal Health Commission) for the questionnaire survey. The sampled CHCs accounted for 20% of the total CHCs, and the subjects account for 10% of the total number of GPs. In total, we obtained 781 valid questionnaires, with a validity rate of 99.0%, after excluding questionnaires in which the age of the GPs was incorrect.

The inclusion criteria for the GPs were: 1) Full-time employed at a CHC that conducts standardized general practitioner training; 2) Willing to participate and answer questions in the questionnaire objectively.

### Methods

We extracted corresponding information from the “2013 Chinese Type 2 Diabetes Prevention Guidelines” [13] and the “Chinese Type 2 Diabetes Prevention Guidelines (Grassroots Edition)” [14] according to six areas (community management goals, screening and diagnosis, key points for community intervention (drug and non-drug), key points for follow-up, and referral criteria) to design 25 questions and formulate the “General Practitioners’ Mastery of Chinese Type 2 Diabetes Prevention Guidelines” questionnaire. The questionnaire was comprised of two sections.

The first section asked about the general status of the practitioners, including sex, educational level, professional title, specialty, position, employment mode, age, years of work experience, and participation in standardized training for GPs.

The second section inquired about the mastery of type 2 diabetes prevention and treatment knowledge in GPs, including the screening of diabetes high-risk population, lifestyle intervention targets, diagnostic criteria, control targets, treatment regimens, and diabetes management to investigate the understanding of GPs of type 2 diabetes prevention and treatment knowledge in China.

### Data collection

An online survey was carried out, and the electronic questionnaire was distributed by WeChat and website hyperlinks to GPs in various communities. A questionnaire contact person was assigned to every CHC to ensure the release and collection of questionnaires. Before data collection, questionnaire execution staff received unified training to ensure that all general practitioners could independently answer the questions and not discuss them with each other.

### Statistical analysis

Excel 2016 and SPSS 24.0 software were used for data analysis. A statistical description was completed for the results of this questionnaire survey. Qualitative data were expressed as frequency (percentage), quantitative data were expressed as (^−^x ± s), data that did not follow a normal distribution were expressed through a median (M) and inter-quartile range (Q1, Q3). For bivariate analysis, the Chi-square test was used for qualitative data, an ANOVA or t-test was used for quantitative data, and the rank-sum test was used when data did not fulfill the conditions for parametric tests. A difference of *P* < 0.05 was considered to be statistically significant. Differences that were statistically significant (*P* < 0.05) in the bivariate analysis were included in the multivariate unconditional logistic regression.

Type 2 diabetes prevention and treatment knowledge mastery was classified based on the number of correct answers. A score higher than the mean was considered to be a passing score; a score lower than the mean was considered to be a failing score [13].

## Results

### General status of GPs

This study included 781 GPs from 54 CHCs in 16 administrative districts in Shanghai. Table 1 shows that sex, age, educational level, specialty, professional title, and other attributes.

**Table 1:** General status of general practitioners

Item	[n (%)]	Item	[n (%)]
Sex		Employment mode	
Male	220 (28.17%)	Formally employed	744 (95.26%)
Female	561 (71.83%)	Contract system	32 (4.10%)
Educational level		Ex-retiree	5 (0.64%)
Associate’s degree or below	69 (8.83%)	Age	
Bachelor’s degree	621 (79.51%)	< 35 years	189 (24.20%)
Master’s degree or above	91 (11.65%)	35–40 years	170 (21.77%)
Specialty		40–45 years	219 (28.04%)
Clinical medicine	689 (88.22%)	> 45 years	203 (25.99%)
Traditional Chinese medicine practitioner	80 (10.24%)	Years of work experience	
Others	12 (1.54%)	< 10 years	191 (24.46%)
Position		10–20 years	304 (38.92%)
General practitioner (including traditional Chinese medicine practitioners)	661 (84.64%)	≥20 years	286 (36.62%)
General medicine team leader	100 (12.80%)	Professional title	
Others	20 (2.56%)	None	14 (1.79%)
Participated in standardized training?		Beginner	125 (16.01%)
Yes	469 (60.05%)	Intermediate	542 (69.40%)
No	312 (39.95%)	Vice-senior and above	100 (12.80%)

### Mastery status comparison of type 2 diabetes prevention and treatment knowledge in standardized-trained/non-standardized-trained GPs

The results of the 25 questions in the type 2 diabetes prevention and treatment knowledge questionnaire by GPs showed that the three questions with the highest accuracy among standardized-trained and non-standardized-trained general practitioners included the blood pressure goal of Type 2 diabetes patients, treatment principles for combining oral diabetes drugs, and monitoring frequency for glycated hemoglobin. In contrast, the three questions with the lowest accuracy were treatment goals for diabetes in elderly people, screening methods for diabetes high-risk population, and aspirin contraindications.

**Fig. 1:**
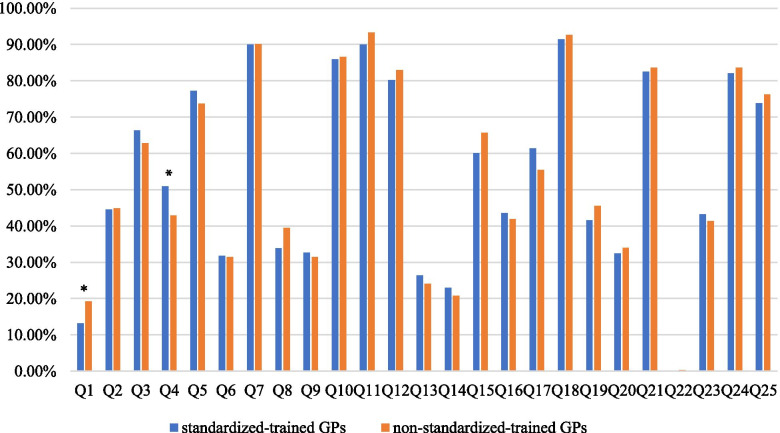
Comparison of Type 2 diabetes prevention and treatment knowledge questionnaire accuracy between standardized-trained/non-standardized-trained general practitioners. *P < 0.05

The statistical analysis results showed that the number of correct answers given by standardized-trained GPs was 13.58 ± 3.31, and the mean accuracy rate was 54.32%. The number of correct answers given by non-standardized-trained GPs was 13.64 ± 2.95, and the mean accuracy rate was 54.56%. The accuracy rates of the 10 questions including the intervention goals for diabetes high-risk population(Q3), the diagnostic criteria for diabetes (Q4), diabetes classification(Q5), the control objective of HbA1c(Q6), the proportion of energy offered by carbohydrate(Q9), the knowledge of intensive insulin regimens scheme(Q13), the contraindications of aspirin(Q14), the first choice of hypertensive drugs(Q16), the therapeutic in diabetes patients with acute coronary syndrome(Q17), and the main aspects of diabetes management(Q23) were higher among standardized-trained GPs than non-standardized-trained GPs, but the difference was not statistically significant. In addition, differential analysis results showed that among the 25 questions, there were significant differences between the standardized-trained and non-standardized-trained GPs for two questions: screening methods for diabetes high-risk population and diagnostic criteria for diabetes (*p* < 0.05). Figure 1 shows the answers given by standardized-trained and non-standardized-trained GPs for all questions.

### Bivariate analysis of mastery of type 2 diabetes prevention and treatment knowledge in GPs

#### Bivariate analysis

Bivariate analysis was used to validate whether sex, educational level, professional title, specialty, position, employment mode, age, years of work experience, and participation in standardized training affect proficiency of type 2 diabetes prevention and treatment knowledge in GPs. The statistical results showed that the mean accuracy rate for all questions was 54.4% and the mean number of correct answers was 13.61 ± 3.17 among GPs. A score of 14 was used as a passing threshold for type 2 diabetes prevention and treatment knowledge mastery; that is, subjects who answered ≥14 questions correctly were considered to have passed, whereas those who answered < 14 questions correctly were considered to have failed.

The proficiency of type 2 diabetes knowledge in GPs (pass or fail) results was used as a results variable, and the Chi-square test was used for differential analysis. The results showed that sex, educational level, and specialty affected the proficiency of type 2 diabetes prevention and treatment knowledge in GPs. The following table shows the bivariate analysis results of various influencing factors (*P* < 0.05, Table 2).

**Table 2:** Bivariate analysis of mastery of type 2 diabetes prevention and treatment knowledge in general practitioners

Influencing factor	Number of subjects who failed [n (%)]	Number of subjects who passed [n (%)]	χ^2^	*P*
Sex			4.038	**0.044**
Male	114 (51.82%)	106 (48.18%)		
Female	246 (43.85%)	315 (56.15%)		
Educational level			27.23	**0.000**
Associate’s degree or below	51 (73.91%)	18 (26.09%)		
Bachelor’s degree	278 (44.77%)	343 (55.23%)		
Master’s degree or above	31 (34.07%)	60 (65.93%)		
Professional title			4.820	0.185
None	10 (71.43%)	4 (28.57%)		
Junior	56 (44.80%)	69 (55.20%)		
Intermediate	253 (46.68%)	289 (53.32%)		
Vice-senior and above	41 (41.00%)	59 (59.00%)		
Specialty			9.198	**0.010**
Clinical medicine	304 (44.12%)	385 (55.88%)		
Traditional Chinese medicine practitioner	49 (61.25%)	31 (38.75%)		
Others	7 (58.33%)	5 (41.67%)		
Position			4.365	0.113
General practitioner	310 (46.90%)	351 (53.10%)		
General medicine team leader	38 (38.00%)	62 (62.00%)		
Others	12 (60.00%)	8 (40.00%)		
Employment mode			1.801	0.406
Formally employed	339 (45.56%)	405 (54.44%)		
Contract system	18 (56.25%)	14 (43.75%)		
Ex-retiree	3 (60.00%)	2 (40.00%)		
Age			5.635	0.131
< 35 years	78 (41.27%)	111 (58.73%)		
35–40 years	78 (45.88%)	92 (54.12%)		
40–45 years	97 (44.29%)	122 (55.71%)		
> 45 years	107 (52.71%)	96 (47.29%)		
Years of work experience			3.490	0.175
< 10 years	77 (40.31%)	114 (59.69%)		
10–20 years	144 (47.37%)	160 (52.63%)		
≥ 20 years	139 (48.60%)	147 (51.40%)		
Participated in standardized training?			0.001	0.978
Yes	216 (46.06%)	253 (53.94%)		
No	144 (46.15%)	168 (53.85%)		

#### Logistic regression of factors affecting mastery of type 2 diabetes prevention and treatment knowledge in GPs

Proficiency of type 2 diabetes knowledge in GPs was used as a dependent variable (a pass was taken to be 1, and a failure was taken to be 0) for multivariate regression analysis of independent variables, which are statistically significant variables from the bivariate analysis. The results showed that sex, educational level, and specialty of GPs were major factors that affected their proficiency in Type 2 diabetes prevention and treatment knowledge (*P* < 0.05). Table 3 shows the specific details.

**Table 3:** Logistical regression of factors affecting mastery of type 2 diabetes prevention knowledge in general practitioners

Factor	Comparison group	Control group	β-value	SE	Wals	*P*-value	OR	95% CI
Sex	Female	Male	0.347	0.165	4.443	0.035	1.415	1.025– 1.954
Educational level	Bachelor’s degree	Associate’s degree or below	1.311	0.289	20.556	0.000	3.710	2.105– 6.538
	Master’s degree or above		2.008	0.369	29.591	0.000	7.447	3.612– 15.352
Specialty	Traditional Chinese medicine practitioner	Clinical medicine	−0.974	0.262	13.832	0.000	0.378	0.226– 0.631
	Others		−0.398	0.624	0.407	0.523	0.671	0.198– 2.283

## Discussion

The results showed that GPs in this study had a better understanding in three aspects of diabetes prevention and treatment knowledge including blood pressure goal of type 2 diabetes patients, treatment principles of choosing the combining for oral diabetes drugs and monitoring frequency for glycated hemoglobin. The questions with a higher accuracy rate among GPs were mainly involved the secondary (complications screening, diabetes diagnosis, and screening of high-risk population) and tertiary prevention (condition monitoring, insulin treatment, and oral glucose-lowering drug treatment) knowledge of type 2 diabetes in China, both of which are frequently used in their daily clinical work. Therefore, this could explain why GPs in this study had better knowledge in these aspects [15–17]. Because GPs in Shanghai mainly manage type 2 diabetes patients other than other types of diabetes, less involved in healthy population disease prevention, therefore they had limited knowledge on treatment goals for special population such as elderly, less experience on screening for high-risk population and proper use of aspirin. Overall, GPs in this study had a satisfactory knowledge of diabetes diagnosis, treatment, classification, and other clinical issues, whereas they had limited knowledge of diabetes prevention. This results indicate that we should provide more resources and training to improve their knowledge and clinical skills on diabetes management and prevention. Bidirectional referral criteria between primary and secondary or tertiary hospitals should be strengthened in GPs [18] to provide a comprehensive diagnosis and treatment regimen for diabetes patients.

We compared the accuracy rates of 25 diabetes prevention questions between GPs with and without standardized-trained, we found that the accuracy rates were identical both in all questions and in the three questions with the highest accuracy rates. The differential analysis showed that only in 2 out of total 25 questions, namely screening methods for diabetes high-risk population and diagnostic criteria for diabetes, had significant differences of accuracy rates between the two groups.

Currently in China, obtaining a GP license requires either undergoing a “5 + 3” (5 years of clinical medicine, including traditional Chinese medicine, undergraduate education, and 3 years of standardized training for general practitioners) standardized training or a transfer training [19]. The training content is formulated according to relevant training outline and is similar in different ways of training [10, 20], and the knowledge that GPs should be competent with is accordingly the same. The goal of standardized training and transfer training is to form a group of primary care physician who can deliver primary health services to individuals, families and communities with standard care. In addition, in order to improve the competency of GPs, continuing education is still needed even after their standardized training or transfer training. The aim of continuing education is to improving the diagnosis and management of diseases and meeting the requirements for handling complex cases during clinical work [21]. In this way, standardized-trained and non-standardized-trained GPs could reach the same level of knowledge and practice skills. Therefore, this explained why our survey showed no difference among these two groups of GPs.

The results also showed that sex, educational levels, and the subspecialty experience prior to becoming a GP were major factors that affected GP’s competency of type 2 diabetes prevention and treatment knowledge. Among these factors, we found that female GPs had better mastery of type 2 diabetes prevention and treatment knowledge than males. Similarly, GPs being specialized in the clinical medicine had better mastery than those being specialized in traditional Chinese medicine. A study showed that female GPs had higher interest in career training and practice and had higher willingness to spend time on in-depth learning [22]. Therefore, female GPs generally have better fundamental knowledge and skills than males. It was revealed that GPs being specialized in the clinical medicine before had better mastery than those being specialized in traditional Chinese medicine. GPs being specialized in the clinical medicine showed better competency on type 2 diabetes consultation and prevention [23].

In addition, the educational level of GPs was also an important factor that affected the competency of type 2 diabetes knowledge. The GPs undergone a higher the educational level showed a greater competency in type 2 diabetes prevention and treatment knowledge. According to Wang et al. [24] on the effects of transfer training in GPs, the educational level affected their training examinations results. In their study, they found that the better the theoretical training results were obtained in GPs had a higher educational level. Similarly, Luo [25] conducted a study to identify the factors influencing the standardized training results of GPs, and it was indicated that the educational level was related to the overall quality of students who participated in training. Students with a higher educational level had a more solid basic knowledge mastery, had a more positive learning attitude and a more complete knowledge system. In contrast, students with lower educational level might have no experience of formal standardized training and with a lower operational literacy, which could correspondingly influence their understanding and absorption of training content. Thus, the students with a lower educational level would present a poorer training results. Furthermore, more chances would be given to GPs with a lower educational level to undergo diabetes-related continuing education and training, which would improve their knowledge and enable them to absorb and master new skills [26].

## Conclusion

Generally, GPs had a better competency in type 2 diabetes diagnostic screening; however, the knowledge in type 2 diabetes prevention and treatment was limited. Factors that affected the mastery of type 2 diabetes knowledge in GPs included sex, educational level, and subspecialty experience. Participation in standardized training was not a major factor that influenced the competency of type 2 diabetes prevention and treatment knowledge, which might be related to the training before obtaining GP license and continuing education after obtaining the license. Standardized-trained and non-standardized-trained GPs in China reach the same level of knowledge and practical skills. The findings also suggested that training of diabetes knowledge and skills should be strengthen for male practitioners, for practitioners with a lower educational level and non-clinical GPs to improve the overall clinical service and to provide standardized care for type 2 diabetes among CHCs.

## Limitations

There were several limitations in this research. Firstly, the questionnaire did not contain every aspect of the disease management although the expert consultation methods were used to ensure the content validity of the questions. Secondly, only the data collected from the submitted questionnaires were analyzed and we didn’t know the further reasons for the significant difference revealed in the results. Maybe an in-depth interview or group discussion should be performed in the future research on the Type 2 diabetes prevention and treatment knowledge mastery for GPs at community health care centers.

## Data Availability

The datasets generated and/or analyzed during the current study are not publicly available as some of the data have not been published but are available from the corresponding author on reasonable request.

## Abbreviations

GPs: General practitioners
CHCs: Community healthcare centers
